# Sciatic Nerve Intrafascicular Injection Induces Neuropathy by Activating the Matrix Modulators MMP-9 and TIMP-1

**DOI:** 10.3389/fphar.2022.859982

**Published:** 2022-05-20

**Authors:** Kuang-Yi Tseng, Hung-Chen Wang, Kai-Feng Cheng, Yi-Hsuan Wang, Lin-Li Chang, Kuang-I Cheng

**Affiliations:** ^1^ Graduate Institute of Clinical Medicine, College of Medicine, Kaohsiung Medical University, Kaohsiung, Taiwan; ^2^ Department of Anesthesiology, Kaohsiung Medical University Hospital, Kaohsiung Medical University, Kaohsiung, Taiwan; ^3^ Department of Neurosurgery, Kaohsiung Chang Gung Memorial Hospital, Chang Gung University College of Medicine, Kaohsiung, Taiwan; ^4^ Department of Microbiology and Immunology, Faculty of Medicine, College of Medicine, Kaohsiung Medical University, Kaohsiung, Taiwan; ^5^ Graduate Institute of Medicine, College of Medicine, Kaohsiung Medical University, Kaohsiung, Taiwan; ^6^ Department of Medical Research, Kaohsiung Medical University Hospital, Kaohsiung Medical University, Kaohsiung, Taiwan

**Keywords:** intrafascicular injection, neuropathic pain, TIMP-1, MMP-9, vascular permeability

## Abstract

**Background:** Peripheral nerve block (PNB) under echo guidance may not prevent intrafascicular anesthetic injection-induced nerve injury. This study investigated whether unintended needle piercing alone, or the intrafascicular nerve injectant could induce neuropathy.

**Methods:** 120 adult male Sprague-Dawley rats were divided into four groups: 1) group S, only the left sciatic nerve was exposed; 2) group InF-P, the left sciatic nerve was exposed and pierced with a 30 G needle; 3) group InF-S, left sciatic nerve was exposed and injected with saline (0.9% NaCl 30 µL); 4) group InF-R, left sciatic nerve was exposed and injected with 0.5% (5 mg/mL, 30 µL) ropivacaine. Behaviors of thermal and mechanical stimuli responses from hindpaws, sciatic nerve vascular permeability and tight junction protein expression, and macrophage infiltration were assessed. Pro-inflammatory cytokine expression and TIMP-1 and MMP-9 activation at the injection site and the swollen, and distal sites of the sciatic nerve were measured by cytokine array, western blotting, and immunofluorescence of POh14 and POD3.

**Results:** Intrafascicular saline and ropivacaine into the sciatic nerve, but not needle piercing alone, significantly induced mechanical allodynia that lasted for seven days. In addition, the prior groups increased vascular permeability and macrophage infiltration, especially in the swollen site of the sciatic nerve. Thermal hypersensitivity was induced and lasted for only 3 days after intrafascicular saline injection. Obvious upregulation of TIMP-1 and MMP-9 on POh6 and POh14 occurred regardless of intrafascicular injection or needle piercing. Compared to the needle piercing group, the ratio of MMP-9/TIMP-1 was significantly higher in the intrafascicular injectant groups at the injected and swollen sites of the sciatic nerve. Although no gross changes in the expressions of tight junction proteins (TJPs) claudin-5 and ZO-1, the TJPs turned to apparent fragmentation and fenestration-like degenerative change in swollen endothelial cells and thickened microvessels.

**Conclusion:** Intrafascicular nerve injection is a distinct mechanism that induces neuropathy. It is likely that the InF nerve injection-induced neuropathy was largely due to dramatic, but transient, increases in enzymatic activities of MMP-9 and activating TIMP-1 in the operated nerves. The changes in enzymatic activities then contributed to certain levels of extracellular matrix degradation, which leads to increases in endoneurial vascular permeability.

## Introduction

Local anesthesia (LA) has advantages over general anesthesia not only with regards to blunting surgery-induced stress responses, but also in decreasing perioperative blood loss, postoperative thromboembolism, and morbidity and mortality rates (Rodgers et al., 2000; Freise and Van Aken, 2011; Hopkins, 2015). Among the various types of LA, peripheral nerve block (PNB) is thought to be superior to central neuraxial anesthesia because it blocks a specific part of the body, limits fluctuation of hemodynamic responses, reduces postoperative pain, and shortens outpatient recovery time (Niraj et al., 2011). However, inadvertently injecting into nerves may result in neuropathy mainly due to unrecognized intrafascicular (InF) LA injections. Although unrecognized nerve injury may induce only transiently significant neurological deficits (Cohen and Gray, 2010; Eker et al., 2010), some patients experience long-term peripheral neuropathy with neurological deficits (Barrington et al., 2009).

The impact of nerve injury-triggered opening of the blood nerve barrier (BNB) not only precipitates endoneurial edema, but also the development of chronic pain (Lim et al., 2014). The migration of macrophages into an injured peripheral nerve is accompanied by an increased expression of MMPs, particularly MMP-9, a gelatinase (MMP-2 and MMP-9) containing a gelatin-binding domain with three fibronectin-like repeats (Siebert et al., 2001; Hughes et al., 2002). Increased MMP may be capable of degrading various protein components of the extracellular matrix, including collagen IV, which is the most abundant component of the basement membrane in the blood-brain barrier (Xu et al., 2019). Although increased MMP-9 secretion has been demonstrated in peripheral nerve injury models (Shubayev et al., 2006; Chattopadhyay et al., 2007; Kawasaki et al., 2008; Remacle et al., 2018), its protein expression and enzyme activity in an intrafascicular-injured sciatic nerve model are unclear. Meanwhile, inflammatory cells around injured sites secrete endogenous tissue inhibitor of metalloproteinases 1 (TIMP-1), which play important roles in regulating MMP activity by inhibiting the MMP proteolysis of the basement membrane proteins for cellular homeostasis, adaptation, and tissue remodeling (Moore and Crocker, 2012; Shubayev and Strongin, 2018).

Pressure-induced blood flow interruption during unrecognized forced injection of LA into the InF space resulted in severe fascicular injury (Hadzic et al., 2004) and axonal degeneration and inflammatory cell infiltrations (Kapur et al., 2007). Our previous study showed that sciatic nerve intrafascicular lidocaine 100 µL induced peripheral neuropathic pain, downregulated Nav1.8, increased ATF-3 expression in the dorsal root ganglion, activated glial cells in the spinal dorsal horn and lots of infiltrated macrophages into injury sites (Cheng et al., 2016). This demonstrates that intrafascicular injection is a type of neuropathic pain model. However, whether an unintended needle piercing only or intrafascicular nerve injectant led to a disruption of the nerve barrier permeability and induced neuropathic pain remains unknown. Therefore, the aim of the present study was to investigate the changes in micro-vessel permeability, expression of inflammatory cytokines, MMP-9, and TIMP-1 in injured sites after intrafascicular injection. Because TIMP-1 binds to MMP-9 to inhibit proteolytic activity, the MMP-9/TIMP-1 ratio was also evaluated to assess its severity after intrafascicular injection.

## Materials and Methods

### Experimental Animals and Groups

**FIGURE 1:**
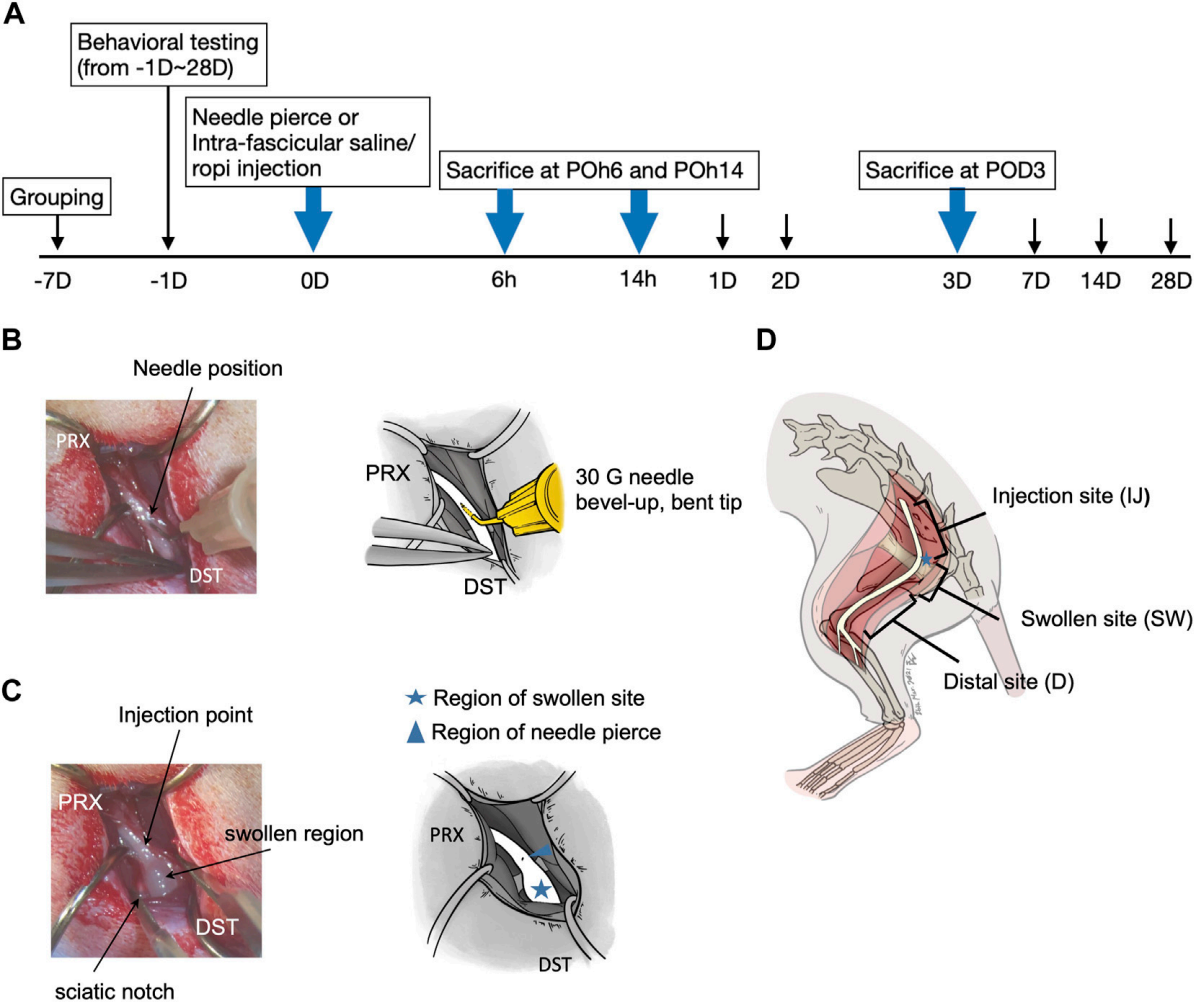
**(A)** Flow chart of the study **(B)** Left sciatic nerve exposed and site with 30 G needle nerve piercing **(C)** Left sciatic nerve exposed with injectates intrafascicular injection (30 µl) **(D)** Sciatic nerves were divided into three sections, injection site (IJ); the length between the proximal cut-off point to about upper margin of the obturator and gemelli muscles, swollen site (SW); the length approximately 10 mm encompassing the notch region, distal site (D); length between the lower margin of the sciatic notch to the cut-off point (distal end before it divided to branches). POh, post-operation-hour; POD, post-operation-day.

All experimental procedures were approved by the Kaohsiung Institutional Animal Care and Use Committee (approval no. 109106). A total of 120 male Sprague-Dawley rats were included in this study. All rats were housed in plastic cages with soft bedding and maintained under a 12-h light-dark cycle (light cycle, 7am–7pm; dark cycle, 7pm–7am), with access to food and water *ad libitum*. The rats were randomly divided into four groups: 1) sham group (control, *n* = 30), left sciatic nerve was exposed only; 2) InF-p group, (*n* = 30), left sciatic nerve was exposed and 30 G needle intrafascicular piercing of the sciatic nerve; 3) InF-S group (*n* = 30), the left sciatic nerve was exposed and injected with 0.9% normal saline (30 µL) at the same designated site along the left sciatic nerve; 4) InF-R group (*n* = 30), left sciatic nerve was exposed, and ropivacaine (30 µL, 0.5%, 5 mg/mL) was injected into the designated site along the left sciatic nerve. Cefazolin (25 mg/kg) was purchased from Sintong Taiwan Biotechnology Co., Ltd., through Kaohsiung Medical University Affiliated Hospital, and freshly prepared in 0.9% saline before use. Ropivacaine hydrochloride (5 mg/mL, AstraZeneca AB, Sodertalje, Sweden) was freshly prepared prior to each intrafascicular injection. The experimental timetable is shown in Figure 1A. The rats were sacrificed at POh6, POh14 and POD3, and the sciatic nerve was removed.

### Surgery Exposure of Sciatic Nerve

All surgical procedures were performed aseptically under isoflurane/O_2_ anesthesia. Each rat was placed in a prone position with the skin of the left hind limb shaved. A linear incision across the skin was made from the region corresponding to the middle point of the ilium along the sciatic notch to the middle of the ischium. The sciatic nerve section along the sciatic notch was identified by blunt separation of the gluteus superficialis from the biceps femoris. Partial dissection of the gluteus superficialis and blunt separation of the gluteus medium and the pyriformis muscles from the sacral region exposed the sciatic nerve from its proximal part under the gluteus medium, nerve at the sciatic notch on obturator and gemelli muscles, to the distal part of the obturator and gemelli muscles. The sciatic nerve was slightly freed from its deeply invested fascia in preparation for subsequent intrafascicular manipulations.

### Intrafascicular Injection and Sample Collection

The left sciatic nerve was exposed, and the point to be pierced with a 30G needle (0.3✕1.3 mm; BD, Franklin Lakes, NJ) was located at 5–10 mm proximal to the upper margin of obturator fascia. To allow for better insertion into the intrafascicular space, the 30G needle was carefully bent with a pair of micro forceps at roughly 30–45 angle about 3 mm from the tip, with the bevel upward and cephalic advancement at the site 5–10 mm proximal to the upper margin of the obturator-gemelli muscles near the sciatic notch (Figure 1B). For either normal saline (0.9%, 30 µL) or ropivacaine (5 mg/mL, 30 µL) intrafascicular injection, the injectant was injected steadily using the aforementioned bent-tip 30G needle with a 1 mL syringe (BD, Holdrege, New England) (Figure 1C). Injection pressure was monitored using a manometer under 15 PSI (pound per square inch, Druck DPI 104; GE Sensing, Druck Limited, Leicester, United Kingdom). For the InF-P group, the bent needle was inserted intrafascicularly in a similar manner without any injection. Sciatic nerves were collected 6 h post-operation (POh6), 14 h post-operation (POh14) and post-operation 3 days (POD3). Sciatic nerves were further divided into three distinct sections: 1) injection site (IJ), the length between sciatic nerve out of ilium bone to proximal part of upper margin of fascia of obturator and gemelli muscles; 2) swollen site (SW), with a length of approximately 10 mm encompassing the notch region, 3) distal site (D), and length between the lower margin of the sciatic notch to the distal end before it divided into branches (Figure 1D).

### Behavioral Responses: Responses to Mechanical and Heat Stimuli

With allocation concealments, hind paw hypersensitivities against mechanical and thermal conditions were assessed as described in our previous work(Cheng et al., 2016). The rats were acclimated to the tested environment for up to 30 min. For mechanical allodynia assessment, the equipment consisted of a metal mesh floor covered by a transparent plastic dome (8 × 8 × 18 cm). A Dynamic Plantar Aesthesiometer (UgoBasile, Italy) with an electronic filament and a maximum cut-off threshold of 50 g was used to measure hind paw withdrawal threshold. The withdrawal threshold was calculated as the average of four to six withdrawal responses to the filament. To measure the latency of hind paw withdrawal from a heat stimulus, each hind paw was set on a glass plate heated by a directed infrared light beam emitted from a moveable light box (UgoBasile Model 7370, Italy). The thermal stimulus was terminated either by withdrawal of the paw from the glass plate or by automation at a 20 s cut-off time. The withdrawal threshold of each paw was calculated as the average of four to six tests, with a minimum of 5 minutes rest between each test.

### Blood Vessel Permeability Assay

Endothelial cells line the blood vessel lumen and form a semi-permeable barrier. Disruption of the endothelial cell barrier can result in increased permeability and vascular leakage. Evans blue albumin (EBA) was used to measure blood vessel integrity and vascular permeability. EBA is a solution of 5% bovine serum albumin mixed with 1% Evans blue dye in sterile 0.9% saline (pH 7.4). EBA was administered surgically under anesthesia via the left femoral vein at a dosage of 1 mL EBA per 100 g body weight and allowed to circulate for an hour. Before the sciatic nerves were harvested, perfusion with fresh sterile saline was performed until no more blue dye was visibly detectable from the outflow. Extravasation of the EBA in the sciatic nerve was observed.

### Protein Extractions and Western Blots

For protein extraction, frozen left sciatic nerve samples were homogenized in a commercially available RIPA buffer (Invitrogen cat. 89,901) containing a complete protease inhibitor mixture (Roche Diagnostics GmbH, Mannheim, Germany). For western blotting, 20 µg of total protein from each sample was loaded onto 10% sodium dodecyl sulfate-polyacrylamide gels (SDS-PAGE) and transferred to polyvinylidene fluoride membranes (PVDF, Millipore, Bedford, MA). The filters were blocked with 5% milk in phosphate-buffered saline (PBS) with 0.1% Tween 20 for 1 h at room temperature and incubated for 24 h at 4 °C with rabbit anti-TIMP-1 (Merck Millipore, AB770), rabbit anti-MMP-9 (Novus Biologicals, NBP2-66955), and mouse anti-rat ß-actin (MilliporeSigma, MAB1501) primary antibodies. This was followed by a reaction with horseradish peroxidase-conjugated mouse anti-rabbit (Santa Cruz Biotechnology, sc-2357) or rabbit anti-mouse secondary antibodies (Santa Cruz Biotechnology, sc-358914) to detect the expression of TIMP-1 and MMP-9 in the IJ, SW, and D sites of the sciatic nerve. The intensity of each band was visualized using ECL western blotting Detection Reagents (Amersham Biosciences, Tokyo, Japan) and captured using the UVP ChemiDoc-It^®^ 810 Imager system (P/N 97–0645-05, 100-115 V–60 Hz, United Kingdom) and ImageJ Analysis System (NIH, Bethesda, MD). TIMP-1 and MMP-9 expression levels were normalized using β-actin. Expression levels were normalized against the levels in the sham control rats.

### Tissue Preparation and Immunofluorescence

The dissected left sciatic nerve tissues were fixed in 4% (w/v) paraformaldehyde and then saturated in 10–30% (w/v) sucrose in 0.02 mol/L PBS (pH 7.4). Once the samples were sufficiently dehydrated in 30% sucrose solutions, they were embedded in an optimal cutting temperature compound (FSC; FSC22 Clear, Surgipath, Leica) in preparation for subsequent cryosectioning procedures. Tissues cut for thin 12-µm cryostat sections were mounted onto glass slides for immunostaining. To detect the expression of ED-1(+) macrophage infiltration, blood nerve barrier (BNB) proteins ZO-1, CLDN5, and cell types expressing TIMP-1 and MMP-9 in sciatic nerve, mouse anti-CD68 monoclonal (BIO-RAD, MCA341GA), mouse anti-RECA-1 monoclonal (Bio-Rad, MCA970GA), rabbit anti-ZO-1 polyclonal (Invitrogen, 40-2300), rabbit anti-Claudin 5 polyclonal (Abcam, ab15106), rabbit anti-TIMP-1 polyclonal (Merck, AB770), rabbit anti-MMP-9 monoclonal (Novus, NBP2-66955), goat anti-GFAP polyclonal (Abcam, ab53554), chicken anti-P0 polyclonal (Novus, NB100-1607), mouse anti-MBP monoclonal (Novus Biologicals, NBP1-05203) primary antibodies were used. After overnight incubation with primary antibodies at 4 °C, Cy3 conjugated goat anti-rabbit (Merck, AP132C), Cy3 conjugated donkey anti-mouse (Merck, AP192C), Cy3 conjugated donkey anti-goat (Abcam, ab6949), Alexa Fluor® 488 conjugated goat anti-chicken (Abcam, ab150169), Alexa Fluor® 488 conjugated donkey anti-rabbit (Abcam, ab150061), and Alexa Fluor® 488 conjugated goat anti-mouse (ab150121) secondary antibody were added for 2 h at 37°C. The stained sections were examined, and images were captured using a ZEISS LSM 700 confocal microscope (ZEISS, Oberkochen, Germany) and a Leica DMi8 inverted microscope fitted with the Leica DFC7000 T fluorescence imaging system.

### Fluorescence Image Analysis

At the beginning of each tissue session, the region of interest (ROI) was manually delineated. In the analyses of sciatic nerve ED-1(+) macrophage intensity, the main ROI consisted of only the endoneurial space outlined by the perineurium. Both types of analyses included three random samplings of background levels for each image. The values for ROI area (Area), total integrated density (IntDen), and mean grey (mean) were recorded for each ROI selection. Fluorescence intensity was calculated as corrected total cell fluorescence (CTCF) based on the procedures outlined in the Open Lab Book. The raw CTCF values were adjusted with their respective ROI areas to correct for differences in the area of selection for each sample on the same slide, ΔCTCF (CTCF/µm). To allow for comparison of relative fluorescence levels of samples between slides, the ΔCTCF^InF^ of each sample was normalized by factoring against the mean relative ΔCTCF^N^ from respective slides. The calculations are summarized as follows.

CTCF=IntDen−(Area x Mean)

ΔCTCF(CTCF/μm)=CTCF÷Area

$$\mathrm{Relative}\;\Delta \mathrm{CTCF}=\Delta \mathrm{CTC}F^{\mathrm{InF}}÷\Delta \mathrm{CTC}F^{N}$$

### Cytokine Array Test

A total of 29 different cytokines and chemokines were tested simultaneously in the IJ, SW, and D sites of sciatic nerve lysates according to the protocol recommended by the manufacturer of cytokine membrane arrays (rat cytokine array, #ARY008, R&D Systems).

### Gelatin Zymography

A total of 25 μg of protein from the IJ, SW, and D sites of sciatic nerve lysates were separated on 10% SDS-PAGE gels containing 0.1% gelatin. After electrophoresis, the gels were renatured with 2.5% Triton X-100 in deionized water for an hour at room temperature to remove SDS. The gels were then equilibrated in developing buffer (50 mM Tris-HCl pH7.8, 0.2M NaCl, 5 mM CaCl_2_, 0.02% v/v Triton X-100) for up to an hour at room temperature, followed by 48-h incubation at 37°C in fresh developing buffer for the development of zymolytic bands.

Gels were stained with Coomassie blue solution at room temperature for an hour, followed by multiple washes in Destaining buffer (10% methanol and 1% acetic acid) for up to 2 hours or until the wash solution was clear. The gels were photographed and processed using the UVP ChemiDoc-It® 810 Imager system (P/N 97–0645-05, 100-115 V–60 Hz, United Kingdom). Alternatively, the gels were carefully wrapped and sealed in clean cellulose paper and allowed to dry. The dried gel plates were then scanned into JPEG images using a flat-bed scanner and analyzed using ImageJ software. Protease bands were detected by the absence of Coomassie Brilliant Blue staining of digested gelatin. Recombinant proMMP‐9 standard mixture (including the mutant MMP‐9ΔOGHem, which lacks the O‐glycosylated and hemopexin domains) was used in each gel as a control. The different protease bands were qualitatively and quantitatively analyzed using the ImageJ TL software.21.

### Statistical Analysis

Time-dependent differences of TIMP-1 and MMP-9 in western blots were determined by ANOVA, followed by the LSD test for multiple post hoc analyses. Group comparisons for behavioral responses and relative ED-1 (+) macrophage fluorescence intensity were made using the Mann-Whitney U test. The SPSS 20.0 (SPSS Inc., Chicago, IL) software was used for all statistical analyses. Statistical significance was set at *p < 0.05, **p < 0.01, and ***p < 0.001.

## Result

In this study, either intrafascicular saline or ropivacaine, at the same volume of 30 µL per an injectant did not induce an injection pressure measured over 15 PSI. However, the injection produced an expanded sciatic nerve swelling along the exposed segment. Symmetric fusiform ballooning of the nerve was easily visible at the sciatic nerve, turning down point on the obturator-gemellus muscles. No rat was reported to be presented as autonomy or exhibited permanent ventroflexion or dragging of the hind paw during forward movement postoperatively. In addition, no rats showed local infection during the investigation.

### Intrafascicular Injection Into Sciatic Nerve Induced Peripheral Neuropathic Pain

**FIGURE 2:**
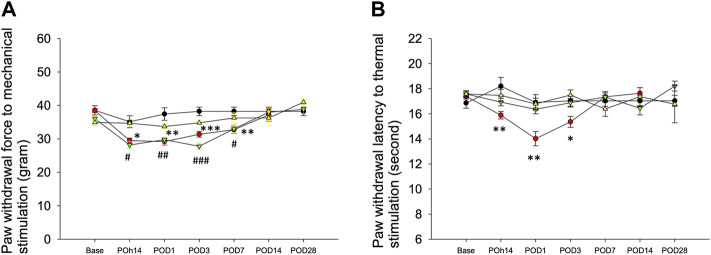
Behavioral responses to mechanical and thermal stimuli. The hind paw withdrawal responses to **(A)** mechanical and **(B)** thermal stimuli after Intrafascicular saline injection (InF-S, red circle), intrafascicular ropivacaine injection (InF-R, green invert triangle) into sciatic nerve or needle pierce sciatic nerve only (InF-P, yellow triangle) were measured. sham group (black circle), removal of left sixth lumbar spine transverse process only was included. There are no significant differences in withdrawal latencies between hind paws in the sham group and needle pierce group. As compared with sham group, intrafascicular saline significantly decreased withdrawal latencies to mechanical and thermal. Intrafascicular ropivacaine induced mechanical hypersensitivity only. POD: postoperative day, n = 30/group, 4–5/each time point. Compare InF-S and sham groups, ***p<0.001; **p<0.01; *p<0.05. Compare InF-R and sham groups, ###*p*<0.001; ##*p*<0.01; #*p*<0.05. Group differences were compared by Mann–Whitney *U*-test. Error bars represent SE.

In comparison with the sham group, behavioral responses were more sensitive to filament withdrawal in the saline or ropivacaine intrafascicular injection groups (Figure 2A). Mechanical allodynia presented as early as POh14, persisted for 1 week (POD7), and then returned to a normal response from POD14 to POD28 (Figure 2A). Intrafascicular saline injection induced thermal hypersensitivity, as indicated by POh14, and lasted for three days (Figure 2B). However, needle piercing did not induce mechanical or thermal hypersensitivity (Figures 2A,B). The results indicated that neuropathic pain was induced regardless of saline or ropivacaine intrafascicular injection.

### Intrafascicular Injection Into Sciatic Nerve Results in Increasing Vascular Permeability

**FIGURE 3:**
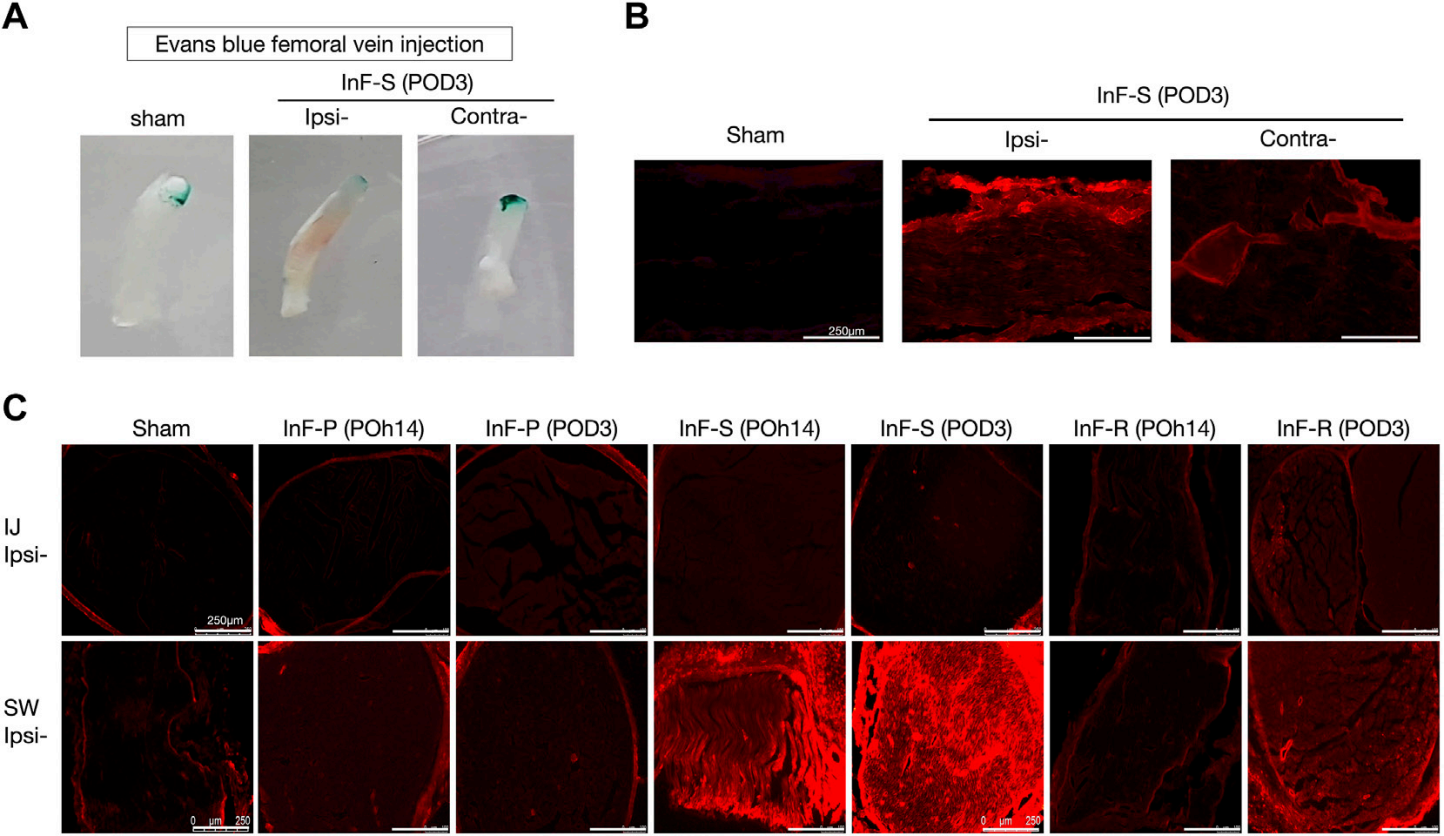
Disruptions of blood nerve barrier integrity in the intrafascicular (InF) injection group. EBA was administered via the left femoral vein at a dosage of 1 mL/100 g. Rats were treated with EBA for 60 min before sacrifice. Following normal saline perfusion, sciatic nerve was collected for histological analyses **(A)** Obvious damage was observed on the ipsilateral (ipsi) sciatic nerve that underwent InF-saline (s) injection. Vascular extravasation of Evans blue-Albumin (EBA) into endoneurial space of InF injection groups’ samples via fluorescence microscopy of **(B)** longitudinal-, and **(C)** cross-section slides.

Blood vessel permeability was assessed using Evans blue test. Intrafascicular saline injection into the sciatic nerve increased vascular permeability and allowed extravasation of Evans blue dye into tissues found on POD3 (Figure 3A). By simple visualization, the healthy endothelium in the sham group prevented extravasation of the dye into the neighboring tissues. The increased permeability on the ipsilateral side of the intrafascicular saline injection group showed significantly increased coloration of the sciatic nerve compared to the contralateral side (Figure 3A). Fluorescence microscopy revealed Evans blue dye fluorescence in the red region of the spectrum, and the sciatic nerve longitudinal sections showed Evans blue dye accumulation on the ipsilateral side (Figure 3B) and especially swollen site (SW site) of the transected sciatic nerve of the intrafascicular saline and intrafascicular ropivacaine injection groups (Figure 3C).

**FIGURE 4:**
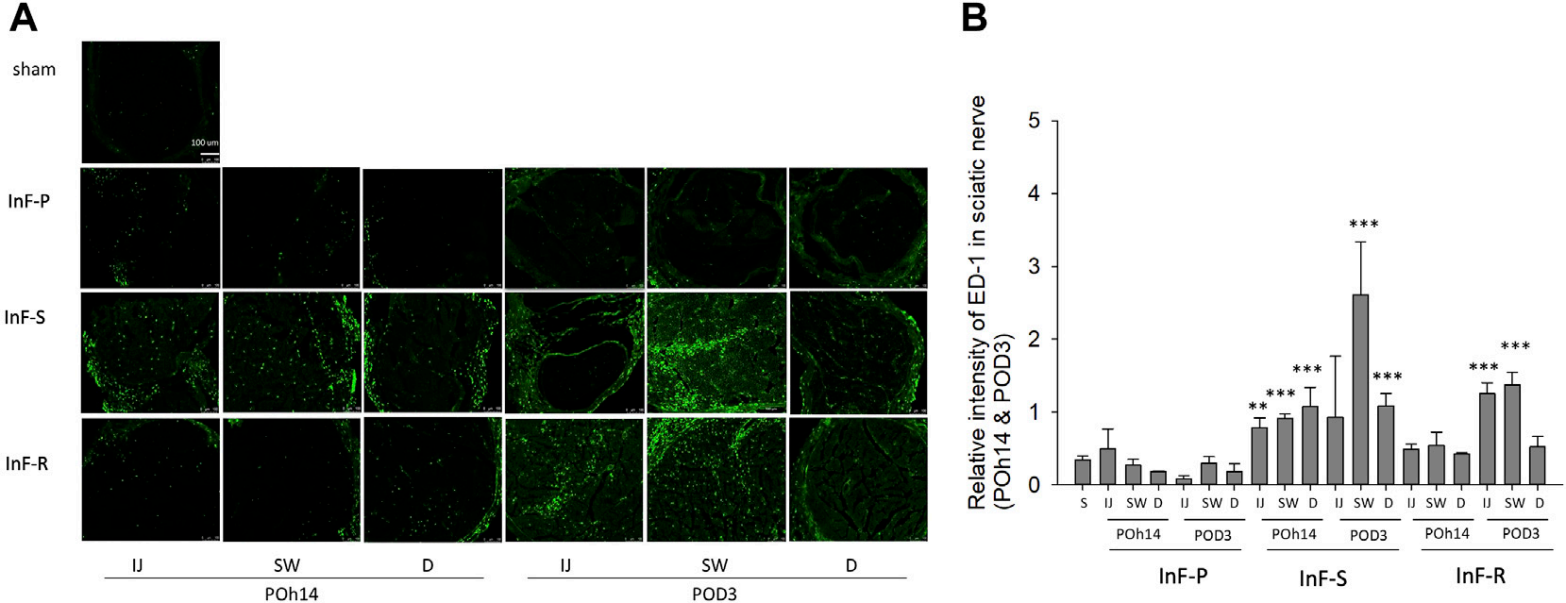
Intrafascicular saline/ropivacaine injection into sciatic nerve increased macrophage infiltration **(A)** Representative immunofluorescent images and **(B)** quantification of ED-1(+) infiltrating macrophage in the three distinct cross-sections of sciatic nerves were showed. Intrafascicular saline (InF-S) injection demonstrated more infiltrating macrophage in the sciatic nerve versus the sham group at POh14 and POD3. Whereas only the IJ and SW sites of sciatic nerve from intrafascicular ropivacaine (InF-R) injection group on POD3 significantly increased ED-1(+) macrophages was found. POD, post-operation-day; injection site (IJ), swollen site (SW), distal site (D) of sciatic nerve. Needle pierce sciatic nerve only (InF-P). Group differences were compared by Mann–Whitney *U*-test. Error bars represent SE. ***p<0.001, **p<0.01. scale bar, 100 µm.

We also found that either intrafascicular saline injection into sciatic nerve in POh14 and POD3 or intrafascicular ropivacaine injection in POD3, but not needle piercing, significantly increased macrophage infiltration in the sciatic nerve (Figure 4). It suggested that intrafascicular injectant injection may damage proximal segment of the sciatic nerve with penetrating and pressure-induced injuries. However, damage may also be induced by ischemic injury in the swollen segment of the sciatic nerve.

### Vascular Permeability Is Induced Through Alteration of TIMP-1 and MMP-9 Expression

**FIGURE 5:**
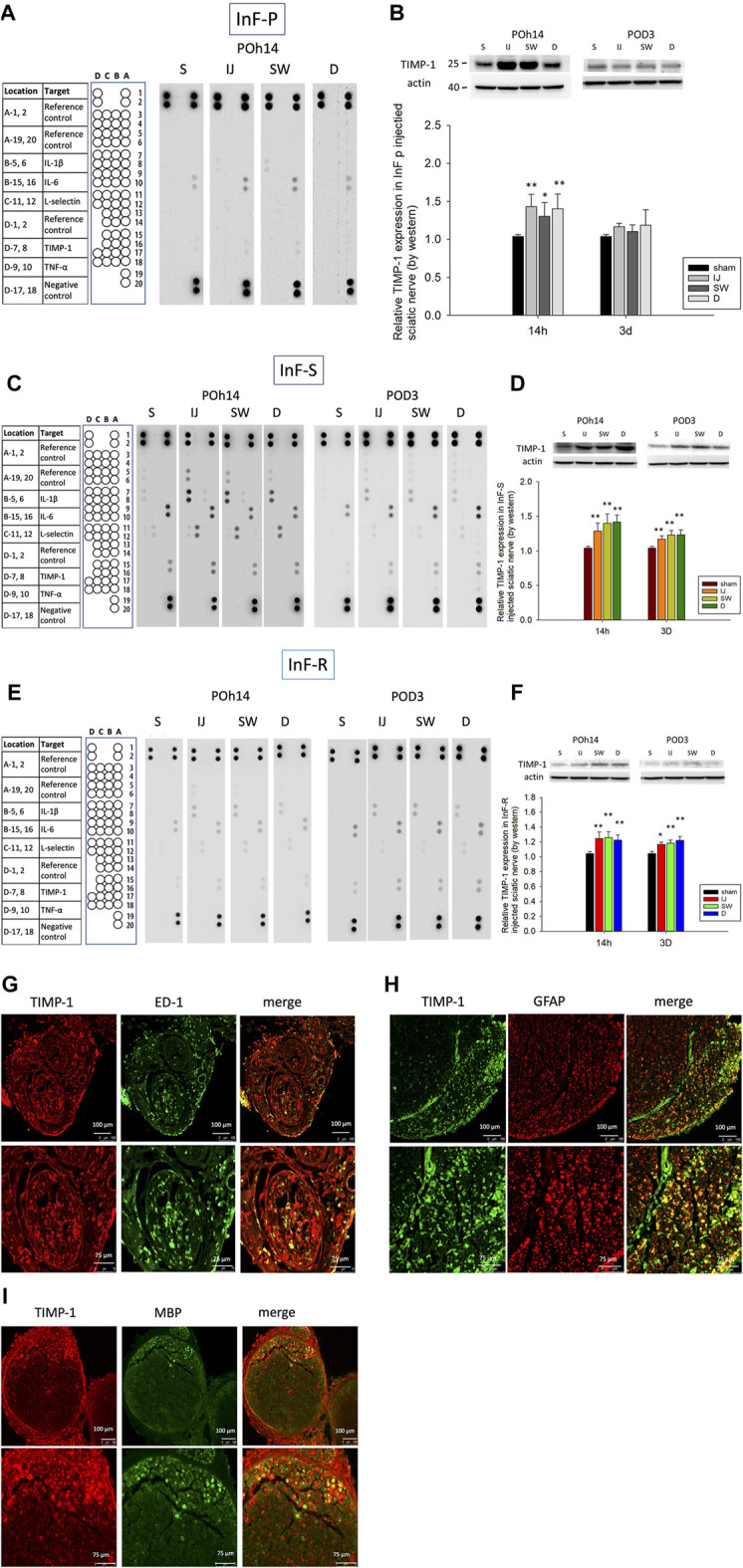
Cytokine expression in three distinct cross-sections of sciatic nerve after intrafascicular injection. Cytokine array was used to measure the expression level of cytokines in sciatic nerve of **(A)** needle piercing group (InF-P) **(C)** intrafascicular saline injection group (InF-S) and **(E)** Intrafascicular ropivacaine injection group (InF-R) at POh14 and POD3. For simple analyte identification of the location of each cytokine, blank rat cytokine array panel coordinate was indicated on the left side of each array. TIMP-1 protein expression level in sciatic nerve were verified via Western blotting in **(B)** needle piercing group (InF-P) **(D)** intrafascicular saline injection group (InF-S) and **(F)** Intrafascicular ropivacaine injection group (InF-R) at POh14 and POD3. Double immunofluorescence staining of TIMP-1 and **(G)** migratory macrophage (ED-1) **(H)** Schwann cell (GFAP) and **(I)** myelin (MBP) in sciatic nerve swollen site in group InF-S at POD3 were shown. Scale bar: 100 and 75 μm. POD, post-operation-day; injection site (IJ), swollen site (SW), distal site (D) of sciatic nerve. S: sciatic nerve of sham group, One-way ANOVA. Error bars represent SE. **p<0.01, *p<0.05., .

Matrix metalloproteinases (MMPs) and tissue inhibitors of MMPs (TIMPs) are crucial for the homeostasis of extracellular matrices. MMPs can affect tight junction proteins, which can influence the integrity of the endothelial barrier. The possible connection between increased permeability and tissue inflammation was also investigated. Cytokine arrays revealed that increased TIMP-1 expression, but not pro-inflammatory cytokines IL-1β, TNF-α, and IL-6, were found in the needle pierce-only group (Figure 5A), intrafascicular saline (Figure 5C), and intrafascicular ropivacaine groups (Figure 5E). Western blotting was performed to detect TIMP-1 protein expression. As compared with the sham group, TIMP-1 was significantly upregulated in the proximal to distal part of the sciatic nerve in the needle pierce group (Figure 5B) and intrafascicular saline/ropivacaine injection groups (Figures 5D,F) on POH14. However, increased TIMP-1 expression in the sciatic nerve lasted for POD3 in the intrafascicular saline/ropivacaine injection groups (Figures 5D,F). Double immunofluorescence staining results demonstrated co-localization of TIMP-1 mainly on migrated ED-1 (+) macrophages (Figure 5G) and unmyelinated Schwann cells (Figure 5H), but not in myelinated Schwann cells (Figure 5I) in the sciatic nerve.

**FIGURE 6:**
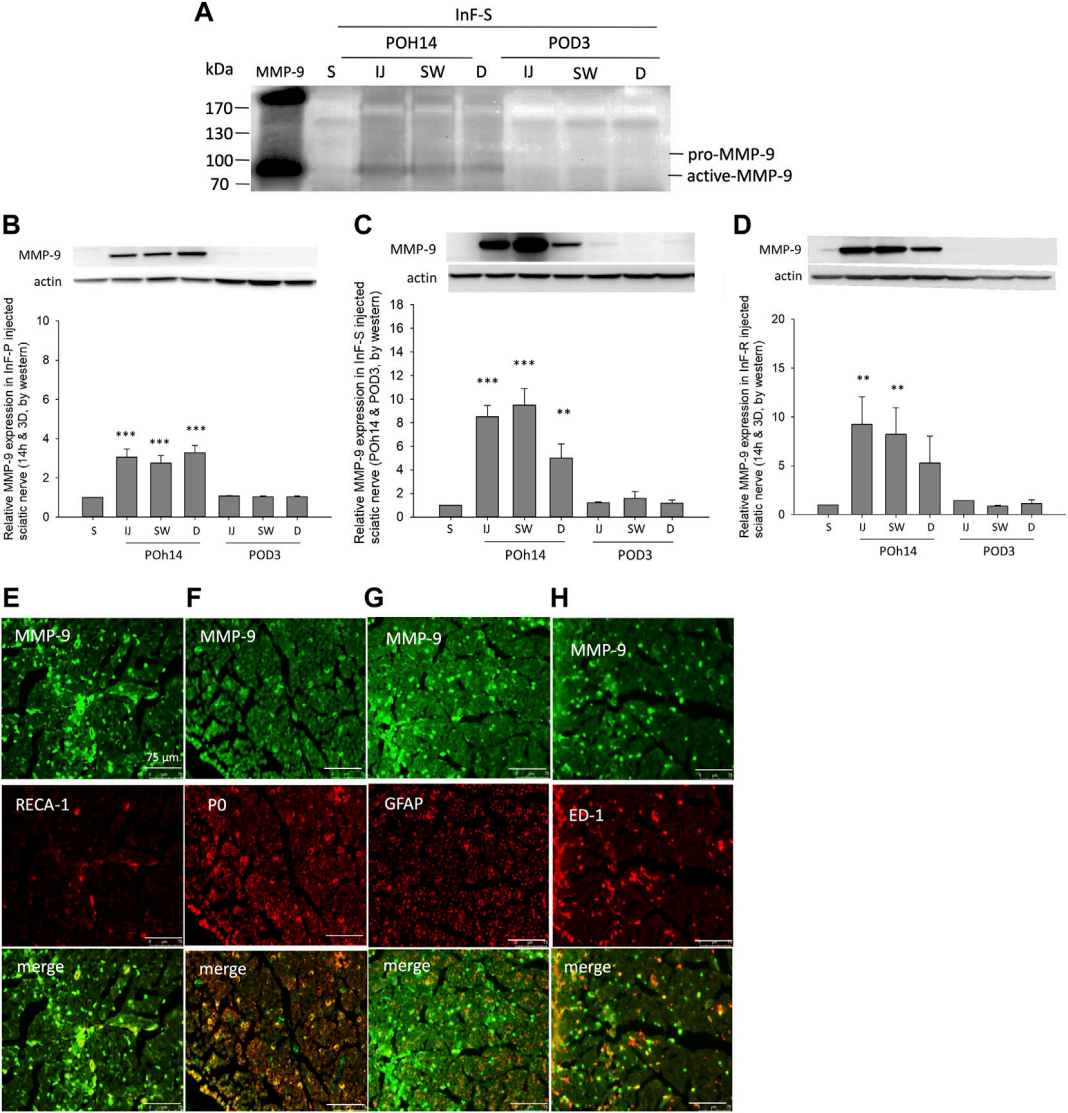
MMP-9 activity and expression after intrafascicular injection **(A)** For gelatin zymography assay, 25 μg of three distinct cross-sections of sciatic nerve from intrafascicular saline group was loaded on the gelatin-containing gels and analyzed. Increased MMP-9 activity was found at POh14. Western blots showed significant increases expression level of MMP-9 in sciatic nerves of **(B)** needle piercing group (InF-P) **(C)** intrafascicular saline injection group (InF-S) and **(D)** Intrafascicular ropivacaine injection group (InF-R) at POh14. Double immunofluorescence staining of MMP-9 with **(E)** RECA-1 **(F)** P0 **(F)** GFAP, and **(G)** ED-1 in sciatic nerve swollen site in group InF-S on POD3 were shown. Scale bar: 75 μm. POD, post-operation-day; injection site (IJ), swollen site (SW), distal site (D) of sciatic nerve. S: sciatic nerve of sham group, One-way ANOVA. Error bars represent SE. ***p<0.001, **p<0.01.

In the *in situ* gelatin zymography assay, MMP-9 activity in the intrafascicular saline injection group was higher in the injected, swollen, and distal sites of the sciatic nerve when compared with the sham group on POh14 (Figure 6A). Consistent results by western blot showed that MMP-9 was dramatically increased on POh14 and then returned to a normal level on POD3 in the needle pierced group (Figure 6B), intrafascicular saline, or ropivacaine injection groups (Figures 6C,D). Co-localization of MMP-9 on vascular endothelial cells (Figure 6E), myelinating Schwann cells (Figure 6F) and macrophage (Figure 6H) but not unmyelinated Schwann cells (Figure 6G) were found by double immunofluorescence.

**FIGURE 7:**
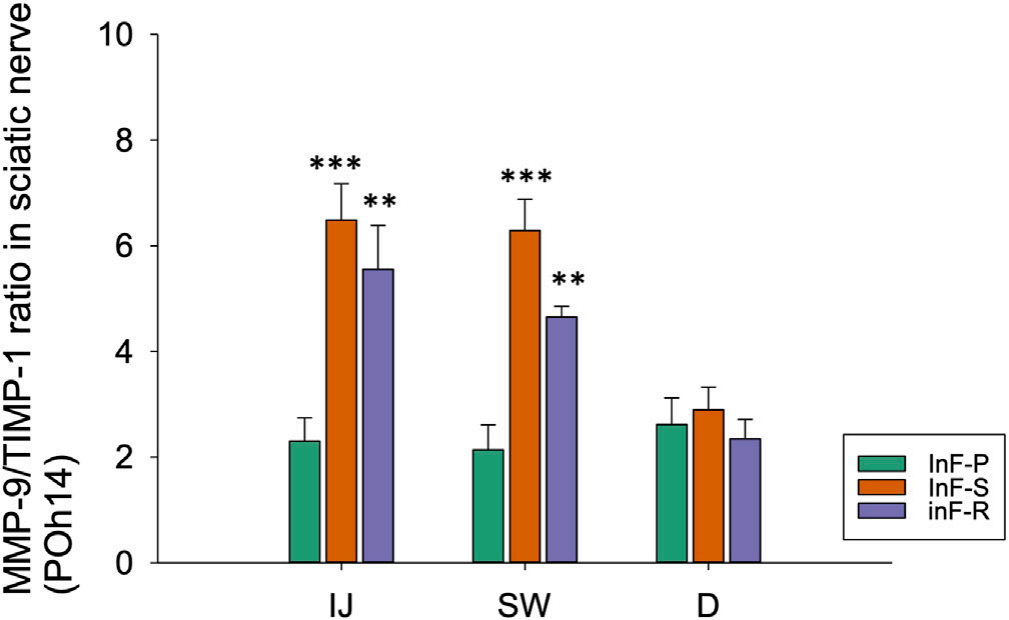
By western blotting, relative expression ratios of MMP-9/TIMP-1 in three distinct cross-sections of sciatic nerve from needle piercing group (InF-P), intrafascicular saline injection group (InF-S) and intrafascicular ropivacaine injection group (InF-R) were shown. The MMP-9/TIMP-1 total protein expression ratio is calculated based on the β-actin normalized value of MMP-9 (Figures 6B–D) and TIMP-1 (Figures 5B,D,F) protein expressions respectively, and relative to the normalized protein expressions in sham samples.

As compared to the needle piercing group, the expression ratio of MMP-9/TIMP-1 in sciatic nerve was significantly higher in the intrafascicular injectant injection groups in the injected and swollen sites of the sciatic nerve (Figure 7). However, compared to the sham group (Supplementary Figure S1A, Supplementary Figure S2A), this study did not observe obvious changes in the expression of tight junction proteins ZO-1 and claudin-5 in either the endothelium or Schwann cells in the proximal injection segment (Supplementary Figure S1B, Supplementary Figure S2B); however, in the swollen segment of the intrafascicular saline group, ZO-1 immunofluorescence presented endoneurial microvessels in fragmented and degenerative changes and claudin-5 condensed expression with swollen endothelial cells (Supplementary Figure S1C, Supplementary Figure S2C). Based on these results, we suggest that increased vascular permeability is associated with increased MMP-9 expression and interruption of the balance between MMPs and TIMPs.

## Discussion

The present study is a unique model that demonstrates unintentional intrafascicular local anesthetic-induced neuropathy. In this study, intrafascicular injection of either saline or ropivacaine induced hypersensitivity of the left hind paw to mechanical stimuli. Although the injectants did not produce pressure over 15 PSI, the needle still broke into the delicate perineurium and damaged the endoneurium directly by needle piercing and volume-occupied induced ischemic injury. In brief, intrafascicular saline or ropivacaine injection facilitates Evans blue leakage, migration of macrophages, increased MMP-9 enzymatic activity, and TIMP-1 secretion on POH14. It is a distinct type of sciatic nerve injury that induces neuropathic pain caused by direct needle piercing and injectant volume expansion.

Intrafascicular saline or ropivacaine injection into the sciatic nerve induced mechanical allodynia, but only intrafascicular saline injection induced thermal hyperalgesia. The neuropathic pain appeared to be induced by the injectants, but not the action of piercing itself. Interestingly, only the saline-injection groups showed significant thermal hyperalgesia, whereas intrafascicular injection of ropivacaine was capable of alleviating the phenotype. This may be closely related to the anti-inflammatory effects of ropivacaine (Hollmann and Durieux, 2000). Ropivacaine possesses anti-inflammatory properties on impaired macrophages by inhibiting granulocyte adherence, migration, and accumulation at the site of inflammation (Cruz et al., 2017). *In vitro* macrophage cell experiments have shown that ropivacaine inhibits the generation of nitric oxide, cytokines, inducible nitric oxide (Boutinguiza et al., 2012), and cyclooxygenase-2 (Wu et al., 2019).

Our previous study revealed that intrafascicular lidocaine administrations induced sciatic nerve neuropathic pain in the hind paw, decreased Nav1.8 expression in the DRG, and activated glial cells in the SDH (Cheng et al., 2016). However, the injectate volume and injectants were discrepant between our present (saline 30 µL, and ropivacaine 30 µL) and the previous studies (lidocaine 100 µL). Small volume intrafascicular saline and ropivacaine still induce nerve injury in light of studies on mechanical allodynia and macrophage infiltration. Although piercing the sciatic nerve alone did not induce obvious neuropathic pain and serious macrophage infiltration, it still activated MMP-9 expression as early as 6-h post-operation (Supplementary Figure S3) and produced a slight neuronal damage in the L4 and L5 DRG (data not shown). This indicated that regardless of the injectant volume, piercing into the intrafascicular segment is sufficient to produce certain levels of nerve injury.

After crush injury of the sciatic nerve, blood-nerve barrier disruption, increased fibrinogen extravasation, and MMP-9 expression may appear as early as 4 h, at its peak level at 24-48 h, and last for 7 days (Liu et al., 2008). In our study, MMP-9 expression increased at POH6 (Supplementary Figure S3), last for POH14 and faded out at POD3 in piercing injury alone and intrafascicular injection-induced volume expanded injury, which was in accordance with the trend of previous studies (Liu et al., 2008; Wu et al., 2020). MMP activation plays an important role in the breakdown of the BNB, subsequent cell recruitment from systemic circulation into the damaged nerve, and development of neuropathic pain (Siebert et al., 2001; Hughes et al., 2002; Ji et al., 2008; Ji et al., 2009). Subsequently, MMP-9 attracted macrophage infiltration to sites of injury from the surrounding blood vessels across the basement membrane, through remodeling of extracellular matrix, upregulation of MMP-9 secretion (Gong et al., 2008; Hanania et al., 2012). Furthermore, MMP-9 degrades the extracellular matrix, including laminin, collagen, and fibronectin, and proteolysis of the basement membrane proteins (Turner and Sharp, 2016; Shubayev and Strongin, 2018) and is an important mediator of blood–brain barrier disruption, edema, and hemorrhage in acute ischemic stroke (Asahi et al., 2001; Switzer et al., 2011).

In the current study, MMP-9 appeared to be secreted from myelinated Schwann cells, endothelial cells, and macrophages following the intrafascicular injections. However, TIMP-1 was mainly secreted from unmyelinated Schwann cells, which preserve the blood-nerve barrier and vascular integrity. Either unmyelinated Schwann cells belonging to the Remak or repair lineage should be investigated further (Jessen et al., 2015). However, these Schwann cells initiated the release of TIMP-1 and then MMP-9 attracted migrated macrophages to injured sites and released TIMP-1 to inhibit the development of inflammatory hypersensitivity. The N-terminal domain of TIMPs binds to the MMP active site with sub-nanomolar affinity and in a 1:1 ratio, inactivating the protease activity of MMP-9 (Brew and Nagase, 2010; Shubayev and Strongin, 2018). Therefore, the MMP-9/TIMP-1 ratio is an important factor in the analysis of the severity of injured nerves. Our present study demonstrated significantly increases in TIMP-1 protein expressions at the injury site within just 6 hours after the intrafascicular saline or ropivacaine nerve injection (Supplementary Figure S4).

Although intrafascicular needle piercing was sufficient to upregulate MMP-9 expression, the TIMP-1 also increased its secretions to alleviate damage to avoid severe enough to increase in vascular permeability, or large amount of macrophage infiltrations. In contrast, InF-S in swollen site with high MMP-9 expression but low TIMP-1 not only induce high levels of MMP-9 expressions, but also severely ischemic damages in swollen site. It resulted in ECM disruption and increases levels of vascular permeability, which further led to high levels of macrophage infiltrations.

The MMP-9/TIMP-1 expression ratio in sciatic nerve tissue in the needle piercing group was significantly lower than that in the intrafascicular saline or ropivacaine injection groups at POH14 in the present study. This indicates that sciatic nerve injury caused by nerve piercing alone is less than that caused by a piercing couple with volume expansion. However, there was no significant difference between the InF-S and InF-R groups.

Due to our intrafascicular injured nerve, MMP-9/TIMP-1 data presentation is different from the crush injury model (Kim et al., 2012) or chronic constriction injury animal model (Remacle et al., 2018). We demonstrated that the intrafascicular injury animal model injured nerves to a slight extent as compared with other nerve injury models, but it is still a novel neuropathy model. Although the injured sciatic nerve resumed back rapidly, a long-term survey of non-myelin (Remak) and repaired Schwann cells in injured nerves are needed to validate anatomical and functional nerve recovery.

Traumatic, ischemic/reperfusion injury, and intrafascicular injury model resulted in an acidic environment and increased MMP-9 secretion through interleukin-1 beta stimulation (Ruhul Amin et al., 2003; Christensen and Shastri, 2015). For cytokine analysis, it has been known that lipopolysaccharide treatment induces pro-inflammatory cytokines TNF-alpha and interleukin-1 beta released within 6 h and rapidly fades out 12 h later in cultured cells(Ghosh et al., 2014; Ye et al., 2017). This could explain why our present study did not show significant TNF-alpha and IL-1ß expression at time interval POh14 in the cytokine array test. However, significantly increases in MMP-9 protein levels in experimental groups were observed as early as 6 hours post-intrafascicular injection in even the needle pierce-only group (Supplementary Figure S3A), as well as the other InF injectant groups (Supplementary Figures S3B, C). The research to assess pro-inflammatory cytokines expression during early post-injury period should be investigated more in further.

In the current study, although no gross changes in the tight junction proteins claudin-5 and ZO-1 were observed. However, fragmentation and degenerative changes in ZO-1 and claudin-5 with swollen endothelial cells and thickened microvessels, especially in the swollen site of the intrafascicular saline injection group, were observed (Supplementary Figures S1C, S2C). This demonstrated that intrafascicular injection injured nerves showed a similar trend to ischemia/reperfusion injury by producing a pressure gradient to inhibit blood flow from volume expansion in the nerve. Peripheral nerve ischemic injury can cause changes in blood flow, nerve conduction block, and BNB disruption (Schmelzer et al., 1989). Our cytokine array results also showed increased L-selectin expression after intrafascicular injection (Figures 5C,E). L-selectin is a member of a family of adhesion receptors that play important roles in lymphocyte-endothelial cell interactions(Ivetic et al., 2019). Therefore, further studies are needed to investigate the morphological changes in tight junctions and adhesion protein expression in intrafascicular injured nerve models to elucidate the causation of increased micro-vessel permeability. Although the present study did not assay for sciatic functional index analysis, previous report from Iohom G. et al. had demonstrated no apparent motor dysfunctions in rats that underwent lateral thigh sciatic nerve intraneural saline or ropivacaine injections regardless of intraneural saline or ropivacaine 0.2 ml injection. (Iohom et al., 2005). Only mild endoneurial fibrosis, axon degeneration, and reduction in Schwann cell density were observed in the two experiment. (Iohom et al., 2005). However, intraneural injection may provide an extrafascicular or intrafascicular injection needs to be validated because the study underwent injection into a sciatic nerve with 3 fascicules at the lateral mid-thigh. However, our research underwent intrafascicular injectant into a uni-fascicle sciatic nerve (proximal part of sciatic nerve). In our current study, this kind of intrafascicular injectants induced sciatic nerve motor dysfunction or not is an interesting issue and should be investigated to clarify it.

In summary, this novel intrafascicular injured nerve animal model mimics clinical practice-induced unintentional nerve injury. Post nerve injury by needle piercing coupled with volume expansion results in neuropathic pain behaviors, increased microvessel permeability by barrier dysfunction, migrated macrophages occupying the injured sites, and rapidly increased remodeling ability with upregulation of TIMP-1 and MMP-9 and their ratio at POh14. However, the long-term recovery of injured sciatic nerves needs to be investigated.

## Data Availability

The original contributions presented in the study are included in the article/Supplementary Material, further inquiries can be directed to the corresponding authors.

## Abbreviations

PNB, Peripheral nerve block; LA, Local anesthesia; S, Sham; InF-P, Intrafascicular pierce; InF-S, Intrafascicular saline injection; InF-R, Intrafascicular ropivacaine injection; POh6, Post operation 6 hours; POh, Post-operation-hour; POD, Post-operation-days; BNB, Blood nerve barrier; EBA, Evans blue albumin; DRG, Dorsal root ganglion; SDH, Spinal dorsal horn; IJ, Injection; SW, Swollen; D, Distal
