# Supplementary figures and images for: Sciatic Nerve Intrafascicular Injection Induces Neuropathy by Activating the Matrix Modulators MMP-9 and TIMP-1
Source: Front Pharmacol. 2022 May 20;13:859982. doi: 10.3389/fphar.2022.859982 (PMC9178525; doi:10.3389/fphar.2022.859982)

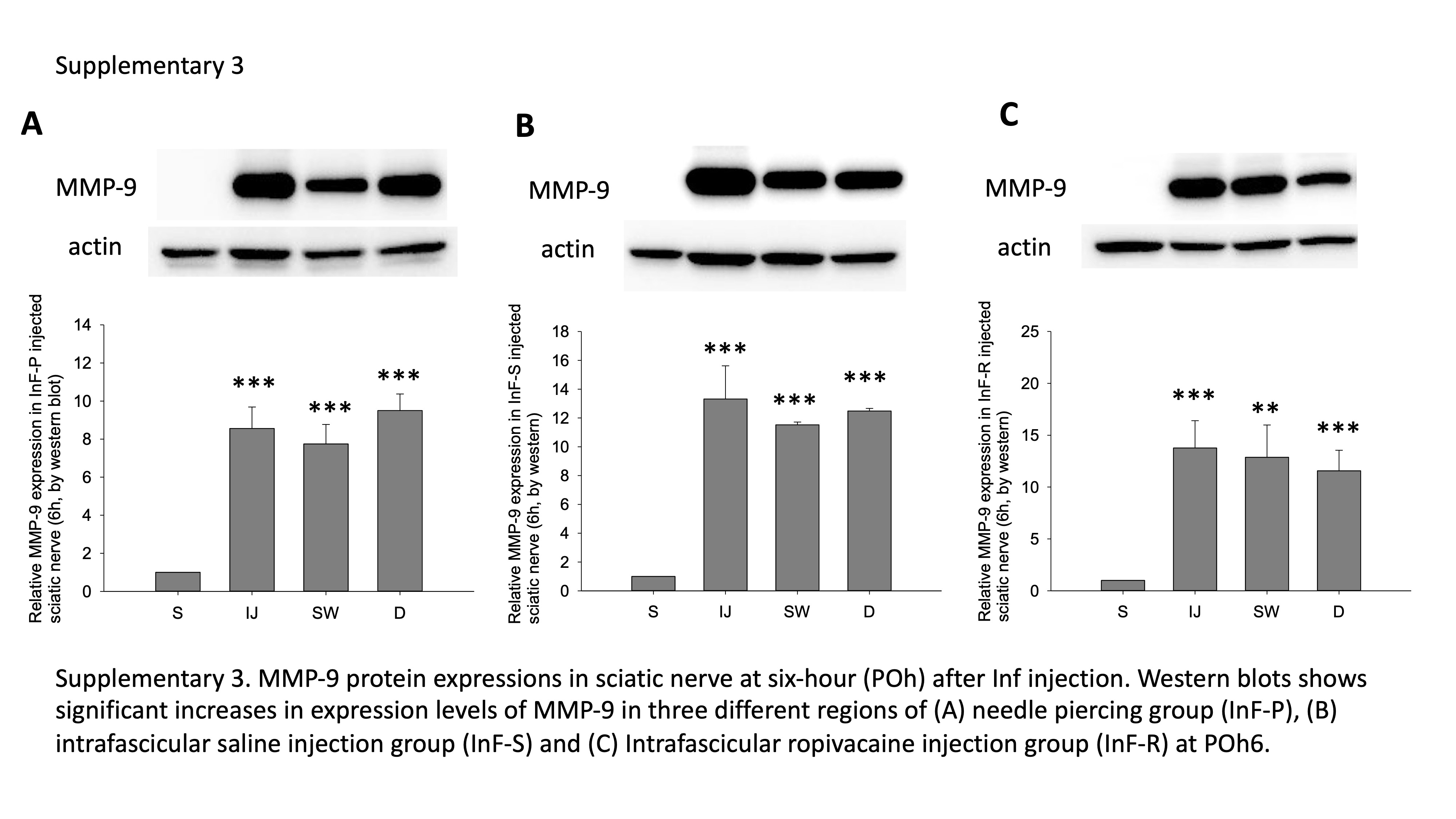

Supplement: Supplementary file 1 [file Image3.JPEG]

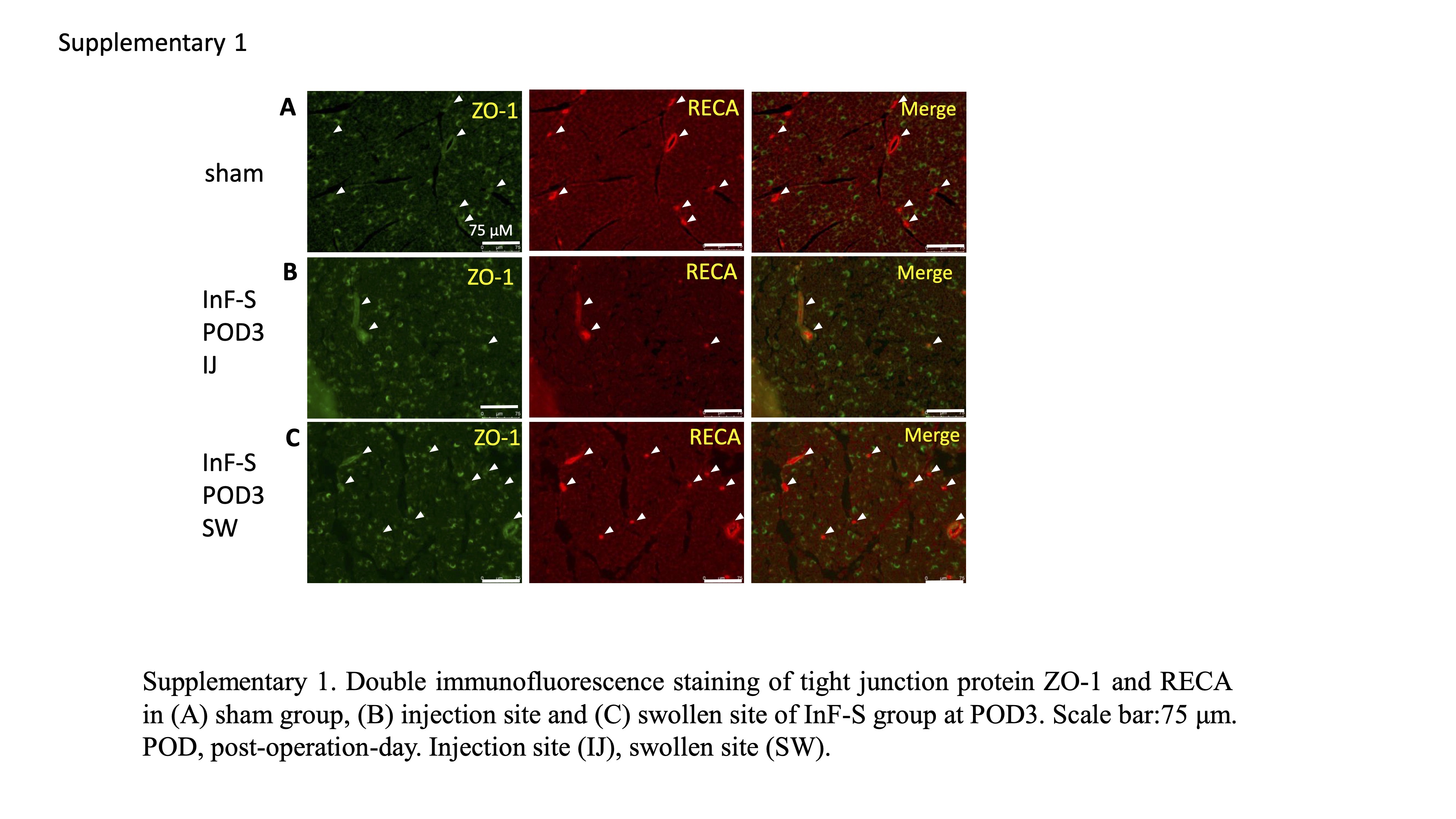

Supplement: Supplementary file 2 [file Image1.JPEG]

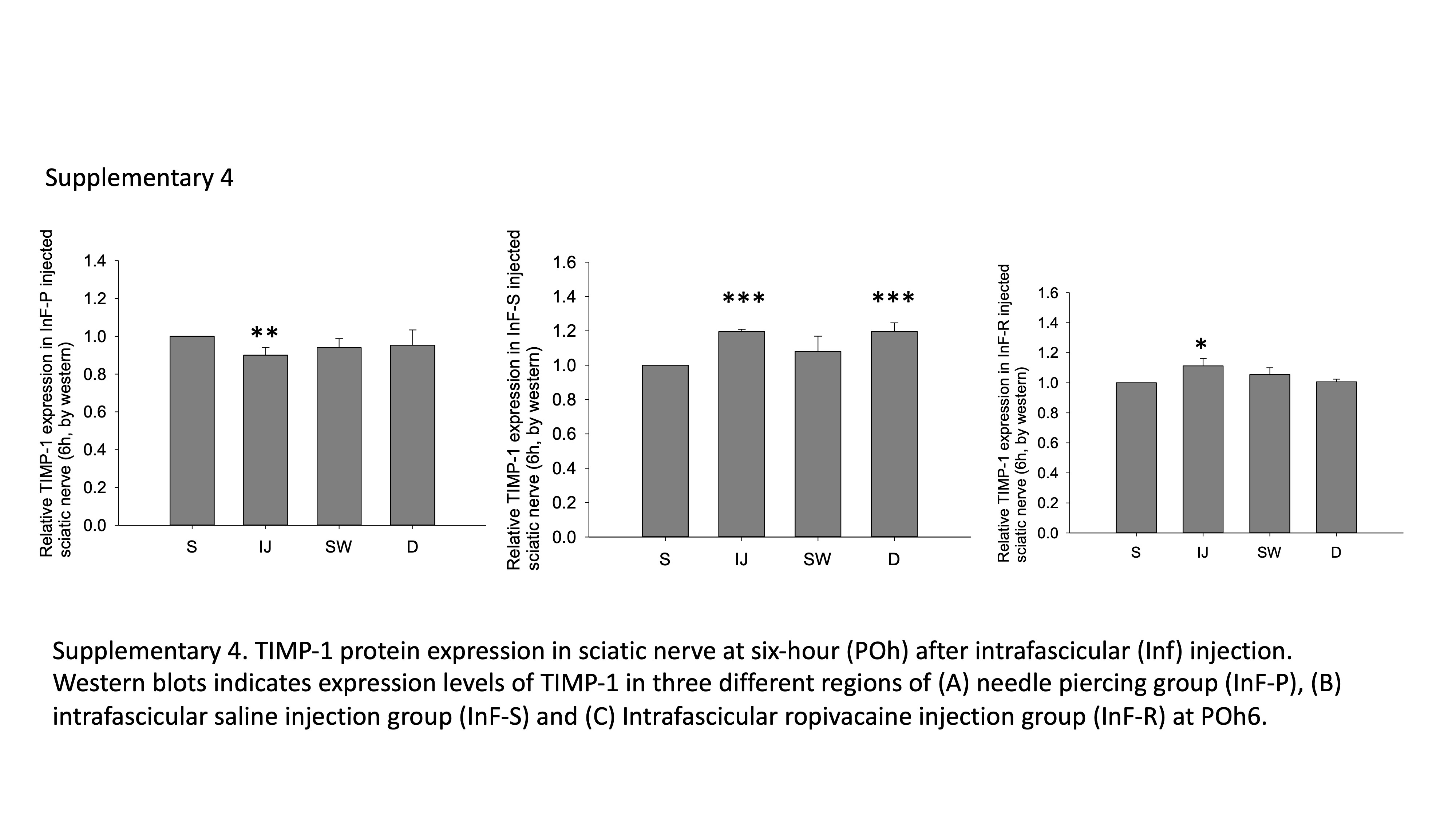

Supplement: Supplementary file 3 [file Image4.JPEG]

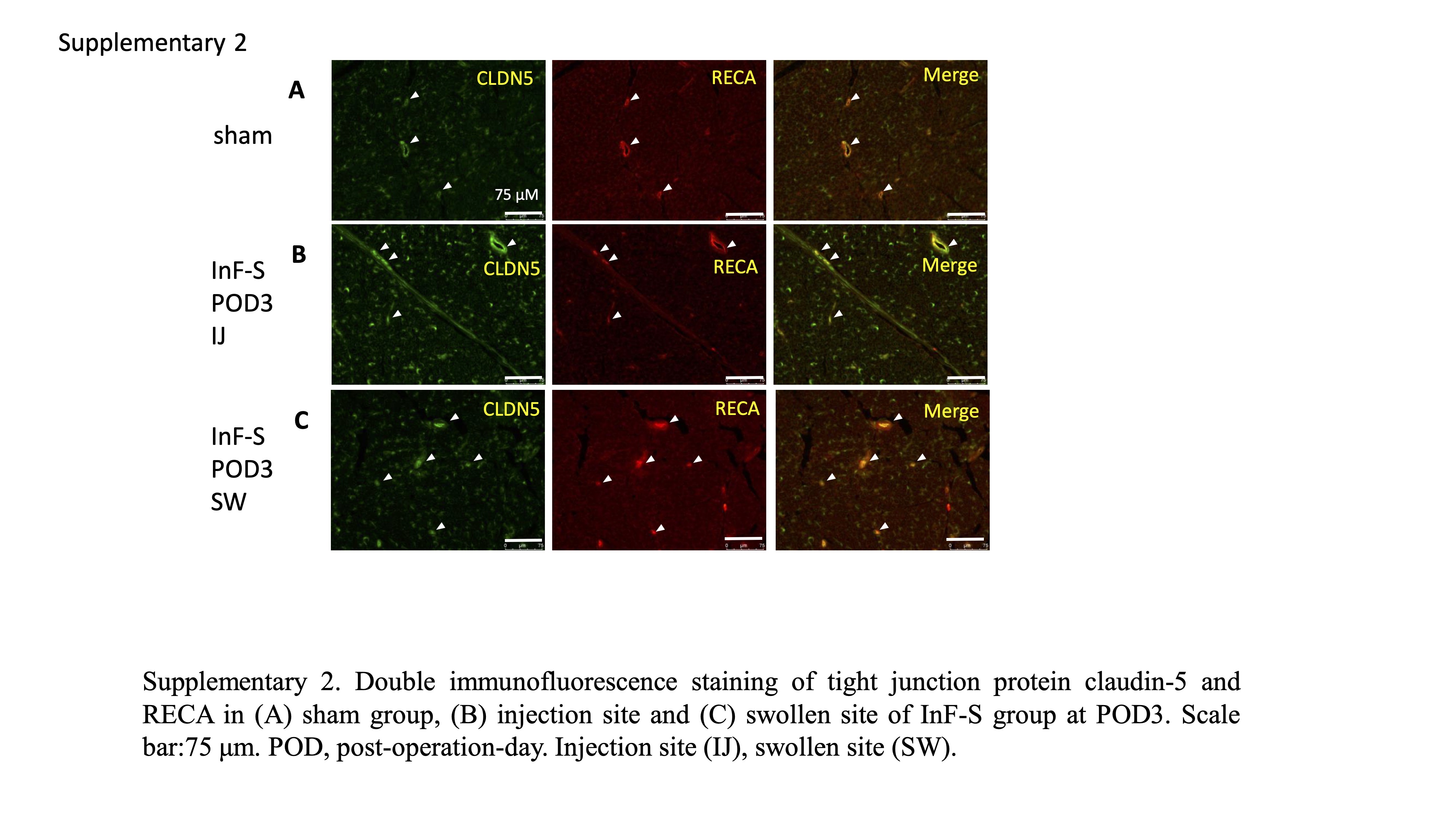

Supplement: Supplementary file 4 [file Image2.JPEG]
